# Biosystematic studies of genus *Withania* Pauquy in Egypt

**DOI:** 10.1038/s41598-024-71500-5

**Published:** 2024-09-18

**Authors:** Faiza A. Shehata, Rim Hamdy, Rehab M. Hafez

**Affiliations:** 1 Botany and Microbiology Department, Faculty of Science, Menoufia University, Shibin El Kom, Egypt; 2 Botany and Microbiology Department, FacultyofScience, Cairo University, Giza, 12613 Egypt

**Keywords:** Anatomy, Morphology, Pollen, Seed, Scot-PCR, SDS-PAGE, *Withania*, Plant cell biology, Plant molecular biology

## Abstract

*Withania* (Solanaceae, Solanoideae) is a widespread genus. Comparative macro-, micro-morphological, anatomical, and molecular features of this genus in Egypt were examined using light and scanning electron microscopy to reassess the conflicted taxonomic relationships between the two studied species. The most significant morphological differences that have been found were: the shape of the lamina, apex, anther, and stigma, and the ratio of calyx tube/lobe; anatomical examination of taxonomic interest are as follows: number of vascular bundles, presence of ears and distribution of accessory vascular bundles in petiole and shape of spongy cells, and number of lower parenchyma in the midrib region of the leaf; trichomes of both species showed no significant differences; pollen, and seed characters are of taxonomic significance in differentiation and characterization between them. Protein profiling revealed that *W. somnifera* has only conserved proteins, while *W. obtusifolia* possessed both conserved and additional proteins in their SDS-PAGE banding patterns. Eleven starts codon-targeted (ScoT) primers were applied and produced 96 amplicons with an average of 70.83% polymorphism/primer. *W. obtusifolia* generated more polymorphic bands and maintained monomorphic ones. SDS-PAGE disclosed that both *Withania* species were 50% related. While Scot-Dendrogram revealed that both *Withania* species were poorly related. So, protein and molecular analyses showed considerable genetic variations between these two species.

## Introduction

Solanaceae is a globally distributed mega-diverse family that includes 98 genera and 2700 species^1–4^, which are distributed throughout the world except for Antarctica. It is represented in the flora of Egypt by 30–33 wild species belonging to eight genera viz. *Datura, Hyoscyamus, Lycium, Nicandra, Nicotiana, Petunia, Physalis, Solanum,* and *Withania*^5–10^. Moreover^11^, reported 25 genera and about 91 species including cultivated species.

*Withania* Pauquy, 1825 is a small genus belonging to the subfamily Solanoideae Kostel, tribe Physaleae D'Arcy subtribe Withaninae contains 10–20 species^3^. It is a naturally occurring plant in drier and more humid environments, spread from the Mediterranean region to South Africa and throughout tropical Africa, as well as from the Cape Verde Islands and the Canary region to Arabia and Middle East regions like India, southern China and Sri Lanka^12^. The generic name is derived from Henry Witham who was a nineteenth-century Scottish palaeobotanist. *Withania* was known to the ancient Egyptians by its fruit which was included in their garlands especially the gorgeous floral collar of Tutankhamun^13^.

At present in Egypt, *Withania* is represented by two species; *W. somnifera* (L.) Dunal^14,15^ and *W. obtusifolia* Tackh.^5–11^. *Withania somnifera* commonly known as Ashwagandha, is considered one of the most precious shrubs in Indian Traditional Systems of Medicine, Ayurveda, and Unani as well as Modern Systems of Medicine^16,17^. All its various parts (leaves, flowers, fruits, seeds, and roots) have been reported to have huge health-promoting activities^18^ such as vitality and fertility of men^19,20^, anti-tumor, anti-inflammation, and anticancer^21^ as well anti-stress effects^22^. It is also used as a tonic, hypotonic, sedative, and diuretic^23^. Some indigenous people of South Africa apply this plant for the treatment of sexually transmitted infections, and asthma and as an anti-inflammatory agent^24^. In Egypt, people widely use the plant in combination with other plants to treat neurological conditions, Alzheimer’s disease, bronchitis, and malaria^25,26^.

Numerous investigations on the morphological^27–29^, anatomical^30–33^, pollen and seed^29,34–40^ traits in some genera of the Solanaceae have shown the use of such information at various taxonomic ranks.

There are several studies on morphology^41–43^, pollen^27,34,44^, seed sculpture^29,44–47^, anatomy^30,48,49^ and trichome^50,51^ of *Withania* species.

The work on *Withania* by^42,52^ remains a major treatment of the genus. According to^13^, the relationship between these two species is so close that many herbarium specimens are difficult to name. In his opinion, the specimens treated as *W. obtusifolia* are just forms of *W. somnifera* with unusually obtuse and long petiolate leaves.

It is extremely difficult to taxonomize a specific genus due to man's interference with selection, cultivation, interspecific hybridization, polyploidization, and the presence of chemotypes, which causes the high divergence of the species' morphology and chemistry^53,54^. Many molecular techniques have been developed as powerful tools for studying genetic relationships and genetic diversity helping the morphological markers to fulfill the accurate identification and discrimination of species^55,56^. Because physiological conditions and environmental stresses rarely affect DNA-based molecular markers, they are used to distinguish genotypes/forms of different genera by detecting DNA polymorphisms^56,57^. Due to its reproducibility, low cost, and powerful ability to reveal polymorphism, start codon targeted (SCoT) analysis received considerable interest based on its universal short conserved region flanking the start codon “ATG”. SDS-PAGE-Protein electrophoresis was also used as a powerful tool for detecting genetic diversity^58,59^.

Therefore, the major goal of this study is to update the earlier knowledge about the *Withania* species in the flora of Egypt based on the revision of materials kept in Egyptian herbaria as well as field studies, the thorough investigation of the macro- and micromorphology and the anatomy of the stem, petiole, and leaf of the genus using light and scanning electron microscopy. In addition, highlighting the evaluation degree of genetic divergence between *W. obtusifolia* and *W. somnifera* using SDS-PAGE and Scot-PCR techniques to gather solid proof regarding whether these forms should be treated as multiple species or as one species with infraspecific taxa for the sake of taxonomy.

## Materials and methods

### Sampling

Fresh materials were collected from natural habitats in Egypt.

Prof. Dr. Rim S. Hamdy, Professor of Taxonomy and Flora in the Department of Botany and Microbiology, Faculty of Science, Cairo University and Member of Cairo University Herbarium performed the formal identification and the comparison of the collected species in the study with authenticated specimens kept in Cairo University Herbarium (CAI) using keys of local flora^6,9^. The studied specimens are arranged in Table 1 according to their phytogeographical territories^60^.

**Table 1:** Collections data of the studied *Withania* species in Egypt.

Withania obtusifolia
**S**: 1930, A. Kaiser 724 (CAI), Wadi Kid, 28° 20.472′N, 034° 10.298ˊE at 655 m elevation, 22–3-2022, B. Simpson s.n. (CAI); 28 ^o^ 20.460ˊ N, 034° 10.331ˊ E at 651 m elevation, 22–3-2022, B. Simpson s.n. (CAI); 28° 20.430′N, 034° 10.427ˊ E at 656 m elevation, Simpson s.n. (CAI). **Ge:** 23–27/1–1929, excursion of the botanical department of Egyptian University, V.& G. T Täckholm s.n. (CAI); 13–2-1932, M. Drar s.n. (CAI), 14–16-2–1933, I.R.Fahmy & M. Hassib s.n. (CAI); W. Kansisrob, 24–10-1956, L. Boulos s.n. (CAI); Bir Kansisrob, 3–2-1962, V. Täckholm, M. Kassas, H. Fawzy, F. Shalaby, M. Samy and M.A.Zahran, 1238 (CAI), Wadi Kansisrob, 17–2-1967, D.J.Osborn & I.Helmy s.n. (CAI); Wadi Kansisrob, 1–2-1979, L. Boulos s.n.(CAI); Khor across the north east slope, 21–1-1962, V. Täckholm, M. Kassas, H. Fawzy, F. Shalaby, M. Samy and M.A. Zahran 160 (CAI); Wadi Aideib, 21–1-1962, V. Täckholm, M. Kassas, H. Fawzy, F. Shalaby, M. Samy and M.A. Zahran s.n. (CAI)
Withania somnifera
**M:** South Rafah, an olive orchard, 12–11-1988, A. G. Fahmy no.1306, (CAI). **S**: Dahab, in a cultivated garden, 22–3-2022, B. Simpson (CAI)
**Nv**: El Faiyum, Beni Salih, 21–11-1926, G. Täckholm s.n. (CAI); Sinnuris District, Ain El-Silien in gardens, 11–11-1982, M. Abd El Ghani 4540 (CAI); Sharqia district, Al gedida village, weed in gardens, 14–5-1978, M. Abd El Ghani 470 (CAI); Shebeen El-kom, Road sides, Menoufia, 5–2022, F. Shehata s.n. (CAI); Cairo University, 6–2022, R. Hamdy & F. Shehata s.n. (CAI); Giza, Ausim, El-Kerateen island, 30° 9 47.5ˊ N, 31° 7 56.2ˊE, 25–7-2022 R. Hamdy s.n. (CAI); Ausim, 30° 9 47.5′ N, 31° 7 56.2ˊE, 25–7-2022, R. Hamdy s.n. (CAI); Qena, Armant-Esna Agriculture road, 16–1-1968, G. Romee & N.El Hadidi s.n. (CAI); 15–4-1977, Chrtek, Kosinova & Slavikova s.n. (CAI). **O**: Kharga Oasis, 6–2-1959, M. Imam s.n. (CAI); Dakhla Oasis, 11–2-1931, M. Hassib s.n. (CAI)

### Macro-morphological investigations

Macro-morphological characters were recorded either directly from about 10–15 fresh specimens before preservation as voucher specimens or from deposited herbarium material, for examination, a binocular stereo light microscope (Leica Wild M3C, Heerbrugg Switzerland) was used. All photographs were taken using a digital camera (Mobile Samsung A 50).

### Pollen and seed micro-morphological investigations

The macro morphological characters of pollen and seeds of about 40 specimens mainly obtained from deposited specimens and fresh material were studied with the aid of a Light-microscope. For scanning electron microscope (SEM), dried samples pollen and seed were mounted on brass stubs and coated with a thin layer of gold using JEOL JSM 530P SEM at an electron microscopic unit, The Applied Center for Insect Nematodes Experimental Station—Giza and a JEOL JSM-IT200 Scanning Electron Microscope (at an accelerating voltage of 20 kV) at the Electron Microscope Unite at Alexandria University, Egypt.

The terminology used to describe the micromorphological characteristics of pollen is consistent with numerous earlier publications^35,61–64^. As for the spermoderm of both species^65–70^, were used.

### Anatomical investigations

Sections of the vegetative organs (stem, petiole, and leaves) were chosen from fresh material. All assessments were made on all plants at similar developmental stages (fruiting stages) and in comparable positions on each plant. Samples were taken from 4th internodes from the apex about 2–3 (cm) and then fixed in FAA (Formalin-glacial acetic acid-70% ethyl alcohol, 5:5:90 V/V). After 24-h fixation, the specimens were transformed into ethyl alcohol series and then embedded in paraffin wax. The specimens were sectioned by a rotary microtome at 10–15 μm; sections were dehydrated in alcohol-xylol series. Sections were stained by safranin and light green according to^71^. The anatomical characters were examined with a Zeiss light stereomicroscope and photographed with a digital camera (OPTIKA). A planimeter was used for the estimation of the percentage of each tissue to the total section area. Terminology according to^72–74^.

### Electrophoresis technique for protein fractionation

#### SDS-PAGE of soluble whole-cell proteins

Protein extractions were performed on 100 mg of frozen tissues collected in triplicate from two *Withania* plants (*W. obtusifolia* and *W. somnifera*). Each sample was ground separately in liquid nitrogen before being mixed with 300 µl of saline and properly vortexed for 30 s. Ten µl of each homogenate was mixed with 20 µl of buffer [10% Sodium Dodecyl Sulfate (SDS), 20% Glycerol, 0.2 M Tris (pH6.6), 10 mM beta-mercapto-ethanol, and 0.05% bromophenolblue] and incubated for 5 min in a 95 °C water bath. Both samples were centrifuged at 13,000 × *g* for 5 min and kept on ice until it is used.

#### Electrophoresis of proteins (PAGE)

The gel was produced according to the method of^75^. It comprised 15% separating gel and 4% stacking gel, respectively. The separating gel is composed of 5 ml (29.2% acrylamide and 0.8% bis-acrylamide), 2.5 ml 1.5 M Tris (pH8.8), 100 µl 10% SDS, 100 µl 10% ammonium persulfate (APS), 100 µl Tetramethylethylenediamine (TEMED) and 2.4 ml distilled water. The stacking gel contained 1.3 ml (29.2% acrylamide and 0.8% bis-acrylamide), 2.5 ml 0.5 M Tris (pH 6.8), 100 µl 10% SDS, 100 µl 10% APS and 100 µl TEMED and 6.1 ml distilled water. BLUeye prestained protein ladder (Cat No. PM007-0500, GeneDireX) with 12 pre-stained proteins of Mwt ranges from 10 to 245 kDa was applied. The two samples and the ladder (180 kDa) were carefully loaded into the wells.

Electrophoresis was accomplished in a vertical slab mold filled with [25 mM Tris–Hcl, 200 mM glycine, and 0.1% (w/v) SDS] running buffer at 80 V for 4 h. After the run, the gel was stained with Coomassie solution for 20 min with agitation to recognize the protein bands. Coomassie solution was composed of 50% distilled water, 40% methanol, 10% glacial acetic acid, and 0.1% Coomassie brilliant blue. The gel was finally destained with a mixture of distilled water, methanol, and glacial acetic acid in a ratio of 5:4:1, respectively, then photographed and stored.

#### Molecular analyses

Whole genomic DNA was extracted from 50 mg of frozen tissues collected in triplicate from the two *Withania* plants using the CTAB (Cetyltrimethylammonium bromide) extraction method of^76^. CTAB buffer comprised 1M Tris HCl (pH 8.0), 5 M NaCl, 0.5 M EDTA, and 20 g CTAB. Polyvinylpyrrolidone and β-mercaptoethanol were freshly mixed into the buffer before extraction. Each tissue was ground separately using liquid nitrogen before mixing with 500 µl CTAB extraction buffer. Each homogenate was incubated for 3 h in a 55 °C water bath. Then, 1.5 µl RNaseA was added to both samples and left for 15 min at 37 °C. At room temperature, chloroform (500 µl) was added to both samples, mix gently, and centrifuged at 16000×*g* for 7 min. Cold ammonium acetate (7.5 M) and cold isopropanol (1:6, v/v) were added to the aqueous phases of both samples. The samples were gently inverted several times, incubated on ice for 30-40 min, and centrifuged at 16000 xg for 3 min. Throw away the supernatant carefully and washed the pellets with 700 µl (70%) ethanol. The tubes were centrifuged at 16000 xg for 1 min, then the supernatants were poured and the pellets were air dried. Finally, the pellets were re-suspended with 50 µl (1×) TE buffer [10 mM Tris-HCl (pH 8.0), 0.1 mM EDTA].

PCR amplifications were performed using eleven start codon-targeted (Scot) primers, Table 2, according to the methods of^76^ with minor modification. In an iced PCR tube, the reaction mixture comprised 12.5 µl DreamTaq Green PCR Master Mix (2×), 2µl primer, and 1µl (50 ng) template DNA forming a 25 μl total volume. The amplification was achieved using Veriti 96-*Well Thermal Cycler*: initial denaturation of 5 min at 95 °C; 40 cycles of 1 min denaturation at 95 °C, 1 min annealing at 56 °C and 2 min extension at 72 °C; and a final elongation step at 72 °C for 10 min. PCR products were separated by electrophoresis (3 hours, 80 v) through 1.5 % (m/v) agarose gel in 1× TAE buffer (40 mM Tris base, 20 mM acetic acid, and 1 mM EDTA, pH 8.0). Band sizes were visualized and evaluated using Gel-Documentation (G: BOX) (SYNGENE model 680XHR, UK) based on a 3000 bp DNA ladder.

**Table 2:** Eleven Scot Primer sequences used in the study.

Primer Name	Sequence 5′–3′
SCOT-13	ACGACATGGCGACCATCG
SCOT-14	ACGACATGGCGACCACGC
SCOT-24	CACCATGGCTACCACCAT
SCOT-31	CCATGGCTACCACCGCCT
SCOT-33	CCATGGCTACCACCGCAG
SCOT-34	ACCATGGCTACCACCGCA
SCOT-52	ACAATGGCTACCACTGCA
SCOT-61	CAACAATGGCTACCACCG
SCOT-66	ACCATGGCTACCAGCGAG
SCOT-70	ACCATGGCTACCAGCGCG
SCOT-71	CCATGGCTACCACCGCCG

#### Protein and DNA analyses

The BioRad Gel documentation system (Image lab V6.1.0 build 7) provided image analysis software for protein and DNA analyses. MvSP software (V3.22) [www.KoVcomp.com] was used to create a phylogenetic tree using the nearest neighbor with Jaccard’s coefficient, and PyElph (V1.3) was used to identify the similarity matrix between the plants by rating the presence (1)/absence (0) of each amplicon.

## Results and discussion

### Macro-morphology

*Withania* Pauquy, Belladone: 14 (1825).

**Fig. 1:**
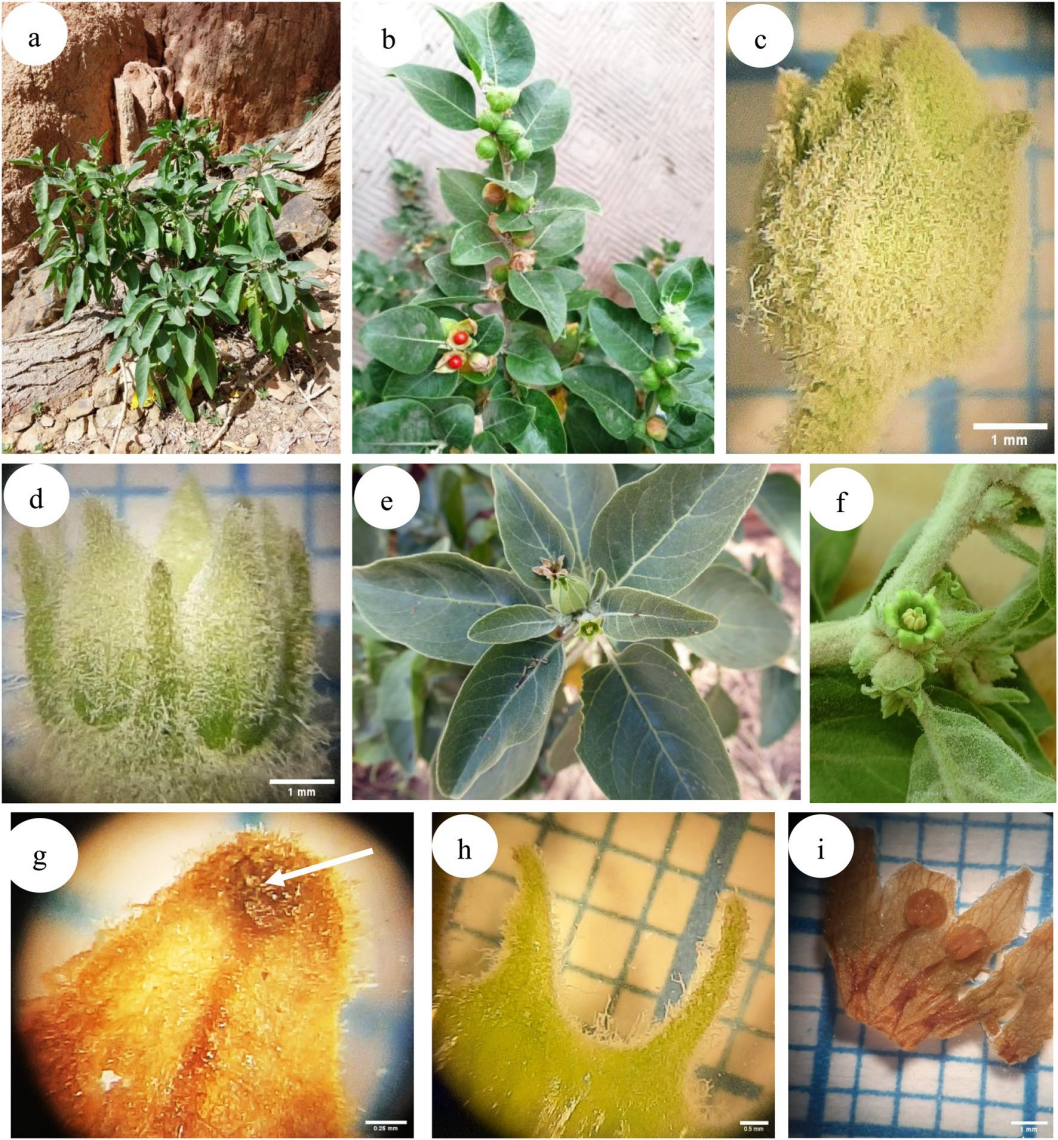

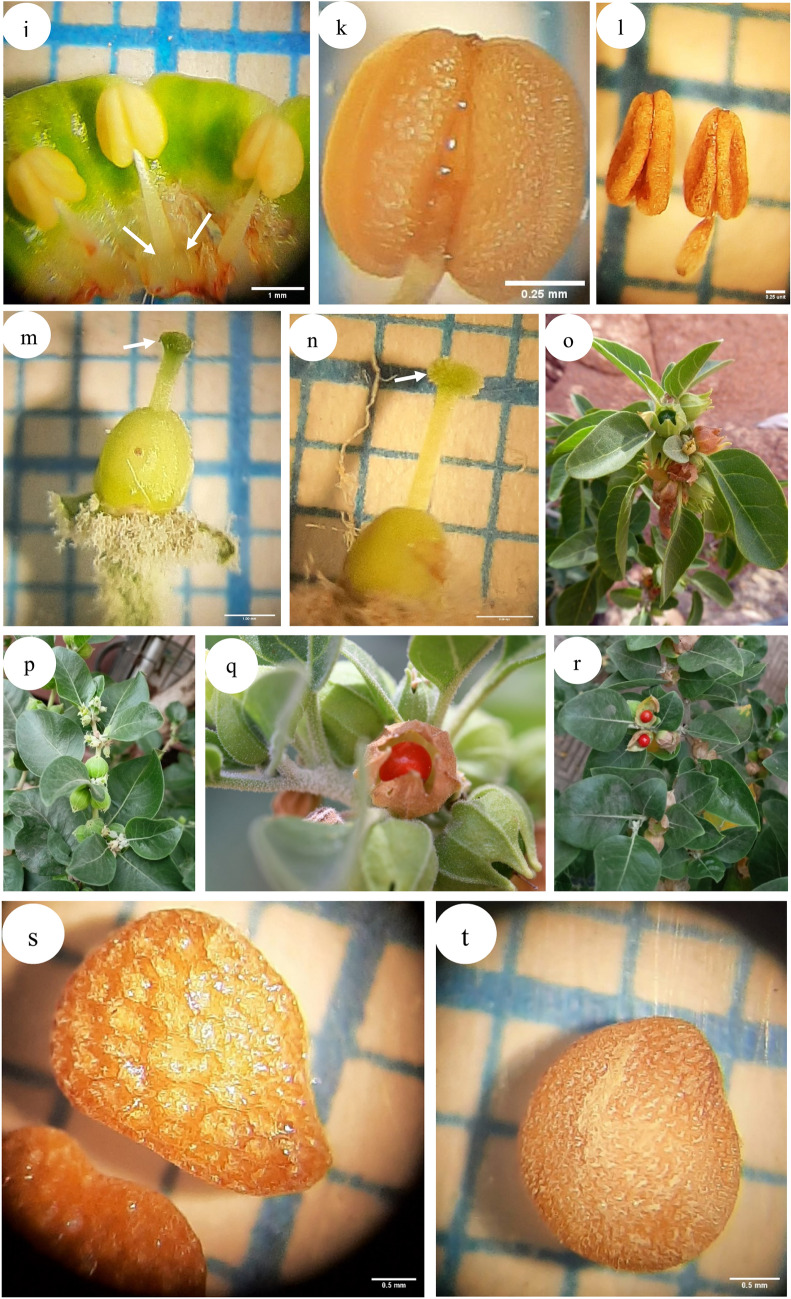
Field photographs showing the morphological characters of *Withania obtusifolia* and *W. somnifera* respectively: (**a**, **b**) Twigs with leaves, flowers and fruits; (**c**, **d**) Flower buds; (**e**, **f**) Flower; (**g**) Sepal deltoid-ovate with a cushion in *W. obtusifolia*; (**h**) Sepal deltoid-lanceolate without a cushion in *W. somnifera*; (**i**) filament base without pillow-like in *W. obtusifolia*; (**j**) filament base with pillow-like in *W. somnifera*; (**k**, **l**) anther; (**m**) gynoecium with clavate stigma in *W. obtusifolia*; (**n**) gynoecium with bi-lobed stigma in *W. somnifera*; (**o**, **p**) plant with immature fruit; (**q**) orange red mature fruit in *W. obtusifolia*; (**r**) bright red mature fruit in *W. somnifera*; (**s**, **t**) 4–5 pitted seed in *W. obtusifolia*; (**t**) one pitted in *W. somnifera.* Photographs of *W. obtusifolia* were taken by Bernadette Simpson and those of *W. somnifera* were taken by Prof. Dr. Rim Hamdy.

Description: Perennial herbs or shrubs with woody texture. Stems are erect, heavily branched with dense hairs. Leaves are solitary or paired, simple, alternate and petiolated. The leaf blade is entire, symmetrical-asymmetrical, hairy or pubescent, often with dendritic hairs. Inflorescences are numerous in congested axillary clusters. The pedicel is short with campanulate and dentate calyx as well as narrowly campanulate, parted to halfway yellowish-green corolla. The stamens are five equal epipetalous, which are inserted near the base of corolla tube. Their filaments are slightly compressed carrying anthers, which sometimes connivent, disc annular surrounded the ovary base. Ovary has 2-locular with numerous ovules and slender style. Fruiting calyx becoming enlarged, surrounding berry, closed at apex. Fruit is globose berry that carries compressed reniform seeds. The significant morphological variations among the two species represented in Egypt are illustrated in Fig. 1 and summarized as follows:

*W. obtusifolia*: Leaf ovate-elliptic with obtuse apex and truncate base. Inflorescence 1–6(8) flowers, calyx with tube longer than lobe, calyx lobe ovate. The Corolla tube is longer than the lobe. Filament without cushions at the base. Stigma clavate. Fruit orange-red with 23–25 seeds and seed diameter 1.8–2.2 × 1.6–2 mm.

*W. somnifera*: Leaf broadly ovate with acute apex and attenuate base. Inflorescence 7–10 flowers, calyx tube shorter than lobe, calyx lobe deltoid-lanceolate. The Corolla tube is shorter than the lobe. Filament with cushions at the base. Stigma bilobed. Fruit bright red with 25–30 seeds and seed diameter (2.3-) 2.5–3 × 1.9–2.5 mm. The detailed morphological variations are listed in Table 3.

**Table 3:** Morphological characters within the studied *Withania* species (significant differences are in italics).

Characters	*W. obtusifolia*	*W. somnifera*
Macro-morphological characters		
Stem		
internodes length (cm)	(1.5)3–7(8)	2.5–6
outline	Cylindrical	cylindrical, except the upper half with ridges
Leaves		
Shape	*elliptic-ovate*	*broadly ovate*
Texture	*Papery*	*thick*
Apex	obtuse	acute-obtuse
Base	± *truncate*	*broadly attenuate*
length (cm)	5–12 (-15)	(3.5-)7–12
width (cm)	(2.8-)3–5.5	(2.5-)4–7
petiole length (cm)	*(1.5-)2.2–4.5*	*(1.2-)1.6–3(-3.5)*
Flower		
pedicel length (mm)	*(3-)4–7*	*2–3(-4)*
Number	*1–6(8), loose*	*7–10, congested*
flower length (mm)	4–5.5(-7)	4–5.3(-5.5)
calyx		
Length (mm)	(2.6-)3–5.9(-6.6)	4.5–5.5(-6.5)
Shape	*deltoid-ovate*	*deltoid- lanceolate*
Lobes	5-fid	5-partite
Tube length (mm)	(1.8)2–3.8(4.1)	2–2.5(3)
Lobe length (mm)	(0.8)1–2.1(2.6)	2.5–3(3.5)
Relation of tube to lobe length	*tube* > *lobe*	*tube* < *lobe*
Lobe apex	Subacute	subacute-obtuse
Presence of cushion at the lobe apex	*Present*	*absent*
Distance between two adjacent calyx lobes	*Obtriangular*	*semicircular*
corolla		
Apex	obtuse-subacute	acute
Colour of veins	*dark green*	*pale green*
Hairiness		
Outer side	*hairy allover*	*basal part- glabrous, dark green upper part-densely hairy*
Inner side	*glabrous*	*hairy*
Length (mm)	4.3–5(7)	4–5.3(5.5)
Tube length (mm)	2.7–3(4.3)	1.8–2.5
Lobe length (mm)	1.6–2(2.7)	2.2–2.8
Relation of tube to lobe length	*tube* > *lobe*	*tube* < *lobe*
Anther		
length × width (mm)	0.6–0.8 × 0.7–0.9	0.6–0.8 × 0.5–0.7
shape	*oblong*	*ovate*
apex	*subacute/apiculate*	*retuse*
Filament		
length (mm)	1.8–2.2(3)	2.2–3
base	*without* two pillow-like on both sides of the filament	*with* two pillow-like on both sides of the filament
gynoecium length (mm)	2–4.5	2.8–3.6
ovary L × W (mm)	1.5- 2 × 1.4–1.5	1.2–1.5 × 0.7–1.4
style length (mm)	1.2–2.3	1.5–2
stigma shape	*clavate*	*bi-lobed*
stigma diameter (mm)	2	1–1.5
Fruit		
peduncle length (mm)	3–7(9)	(2.5)3–4.5
number of fruits	1–4(8)	(3)5–10
colour	orange red	bright red
L × W (mm)	*16–19* × *10–15*	*15–18* × *5–11*
fruiting calyx length (mm)	13–15	12–15
fruiting calyx lobe length (mm)	*3–4*	*2.5–3*
number of seeds/fruit	*23–25*	*25–30*
Seed		
shape	sub-reniform	reniform
length × width (mm)	1.8–2.2 × 1.6–2	(2.3-)2.5–3 × 1.9–2.5
Micro-morphological characters		
Pollen characters		
polar diameter (μm)	(18-)20.67–24.9	23–37.17
equatorial diameter (μm)	19–25.67	17.7–19.13
polar/equatorial ratio	0.95–0.97	1.3–2.09
pollen shape	*subspheroidal*	*prolate*
shape in polar view	spheroidal	spheroidal
shape in equatorial view	*spheroidal*	*oblong*
aperture class	*tricolporate*	*tri-tetracolporate*
exine surface	striate- rugulate	striate-rugulate
ornamentation	foveolate	foveolate
Colpus		
length (μm)	15–20	19–30 (-31)
width (μm)	1–1.667	0.94–1.38
length/width ratio	9–20	14.5–31.3
Bridge		
presence	present	present/absent
position	middle	middle/lateral
length × width (μm)	55–57 × 16–17	6–7 × 3–4
Seed characters		
shape	sub-reniform	reniform
length × width (mm)	(1.6)1.8–2.2 × 1.6–2(× 10^3^)	(2.3-)2.5–3 × 1.9–2.5 (× 10^3^)
length/width ratio	1–1.1	1.2–1.3
Hilum		
shape	*elliptic*	*slit-like*
position	Apical	apical
level	*Shallow slightly protruding*	*deep protruding*
length × width (μm)	441–455 × 68–70	400–410 × 60–62
gross seed coat sculpture	reticulate	Reticulate
epidermal cell shape	polygonal, 7–10-gonal iso-radially elongated	polygonal, 5–6-gonal iso-tangentially elongated
Anticlinal cell wall		
shape	± undulate	± undulate
relief of cell boundaries	raised	Raised
distal appendage	slender ribbon-like finely papillate at the summit of the cell	broad ribbon-like densely papillate
thickness(μm)	25–45.5	28.5–43
curvature of outer periclinal wall	concave with characteristic holes though the bottom thickening	concave thickenings covering the bottom area
lateral wall ornamentation	*Without fibrils*	*With fibrils*

**Fig. 2:**
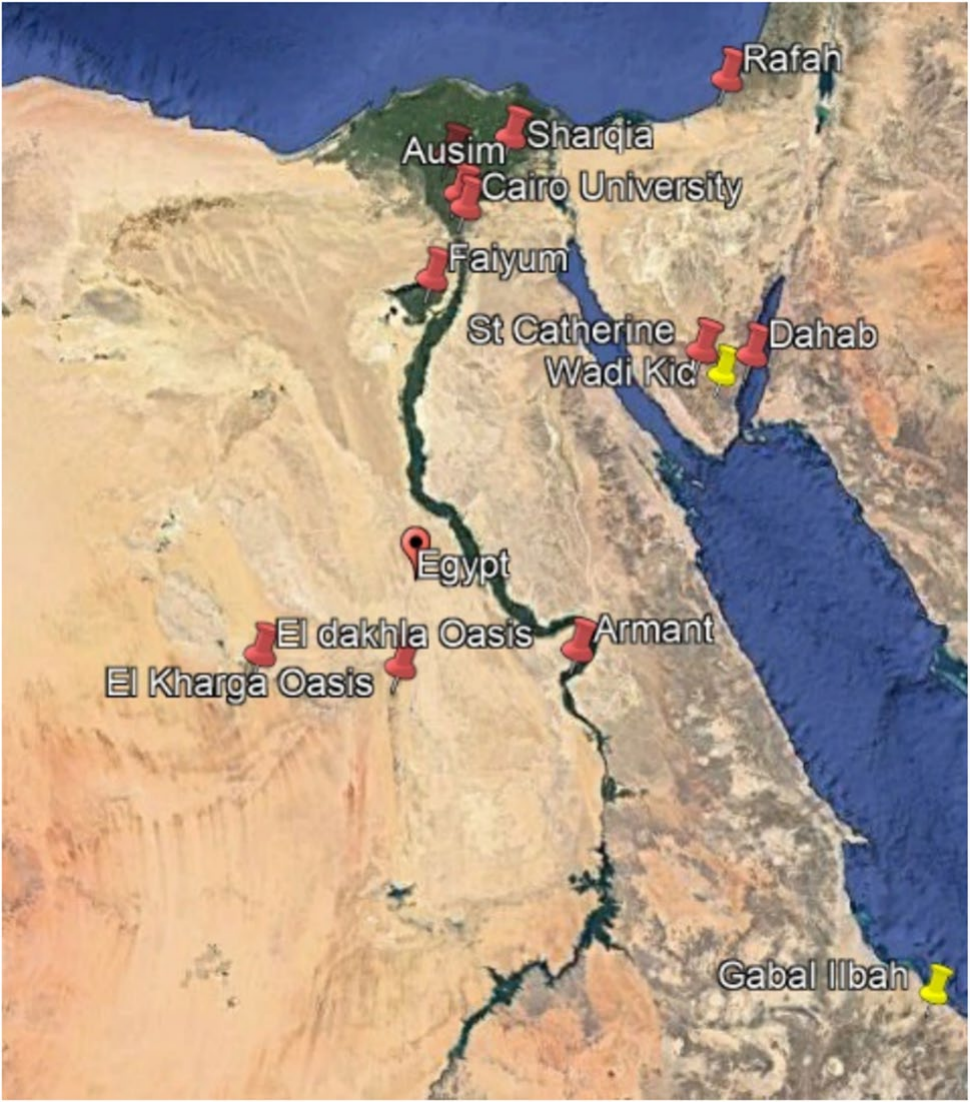
Distribution map of *Withania* species in Egypt showing specimens of *W. obtusifolia* and *W. somnifera* examined by the authors.

Distribution in Egypt: *W obtusifolia*: Sinai and Gebel Elba, *W. somnifera*: Sinai, Isthmic desert, Nile land bordering Nile Delta and Nile Valley and Oasis, Fig. 2.

The morphological variations such as the shape of the leaf and the number of seeds were reported between *W. obtusifolia* and *W. somnifera* by^41,77^. The current investigation validated their results; leaf ovate-elliptic with obtuse apex and truncate base in *W. obtusifolia* while leaf broadly ovate with acute apex and attenuate base in *W. somnifera,* each berry has 23–25 seeds in *W. obtusifolia* and 25–30 seeds in *W. somnifera.* In addition to the previously mentioned feature, there are newly employed characters to distinguish between the *Withania* species under study, such as calyx lobe shape; ovate in *W. obtusifolia* even though deltoid-lanceolate in *W. somnifera,* calyx tube/lobe ratio; tube longer than lobe in *W. obtusifolia* while tube shorter than lobe in *W. somnifera*, hairiness of the interior side of the petal lobe; smooth in *W. obtusifolia* while hairy in *W. somnifera*, filament without cushions at the base in *W. obtusifolia,* while with cushions in *W. somnifera,* anther oblong in *W. obtusifolia*, ovate in *W. somnifera* and seed; 1.8–2.2 × 1.6–2 mm diameter in *W. obtusifolia* while (2.3-) 2.5–3 × 1.9–2.5 mm in *W. somnifera*. Ultimately, the morphological characteristics under examination are distinguishable and diagnostic between the *Withania* species under study.

### Micro-morphology

#### Pollen

Pollen features through LM and SEM are a good source of taxonomic information that can help the species and genera delimitation, and identification and strengthen their systematic position^78^, usually tricolporate rarely 4-colporate in Solanaceae^47^*.*

**Fig. 3:**
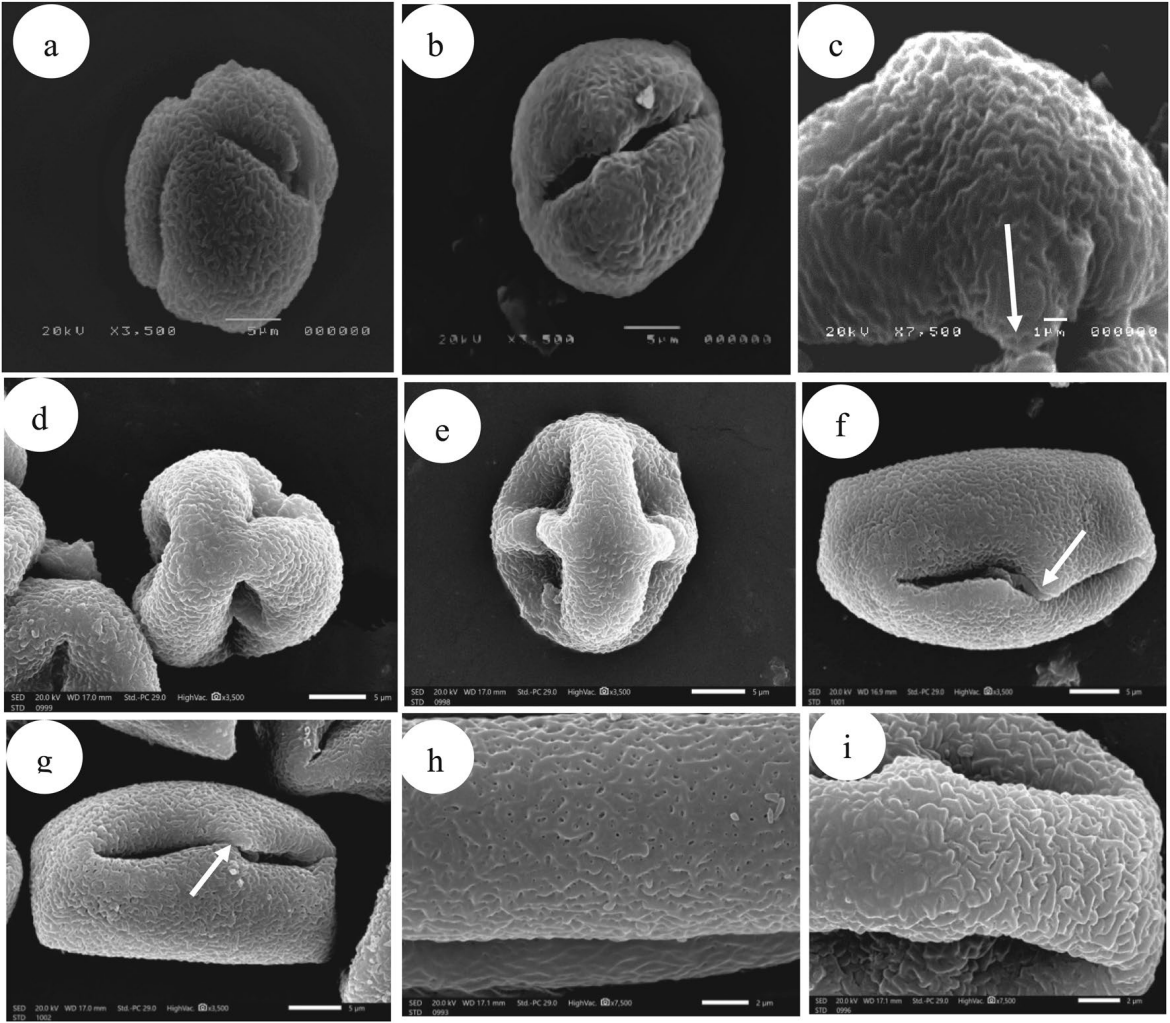
SEM photomicrographs of *Withania* pollen grains: (**a-c**) *Withania obtusifolia*: (**a**) polar view, (**b**) equatorial view, (**c**) showing the bridge and exine ornamentation, (**d-i**) *W. somnifera*: (**d, e**) polar view, (**f, g**) equatorial view showing the bridge; (**h, i**) exine ornamentation.

In *W. obtusifolia*, the pollen class type is tricolporate, sub-spheroidal in shape, with a bridge in the middle of the colpus, while in *W. somnifera*, the pollen class type is tricolporate and tetracolporate, prolate in shape, with or without a bridge in it, and the bridge at the middle or lateral position (Table 3 and Fig. 3).

Pollen morphology of the studied species shows considerable variation in exine pattern which agreed with^34^. Significant pollen characteristics are important in differentiating the investigated *Withania* species; pollen shape subspheroidal in *W. obtusifolia* even though prolate in *W. somnifera,* shape in equatorial view; spheroidal in *W. obtusifolia* while oblong in *W. somnifera,* colpus length/width ratio; 9–20 in *W. obtusifolia* while 14.5–31.3 in *W. somnifera.* Additional characteristics, such as polar and equatorial diameter, shape in polar view, exine surface, bridge prescience and position, reveal overlaps between the species under study.

#### Seeds

The seed characters of the genus *Withania* were investigated using SEM. In both species, the seed shape is quite stable, reniform to sub-reniform with apical not protruding concave (sunken) hilum. The gross overall seed coat sculpture is reticulate. The shape of epidermal cells is irregular and not isodiametric, the cells near the margin are smaller than the others. The walls of anticlinal cells are broadly and evenly thickened, with a sinuate cell margin.

**Fig. 4:**
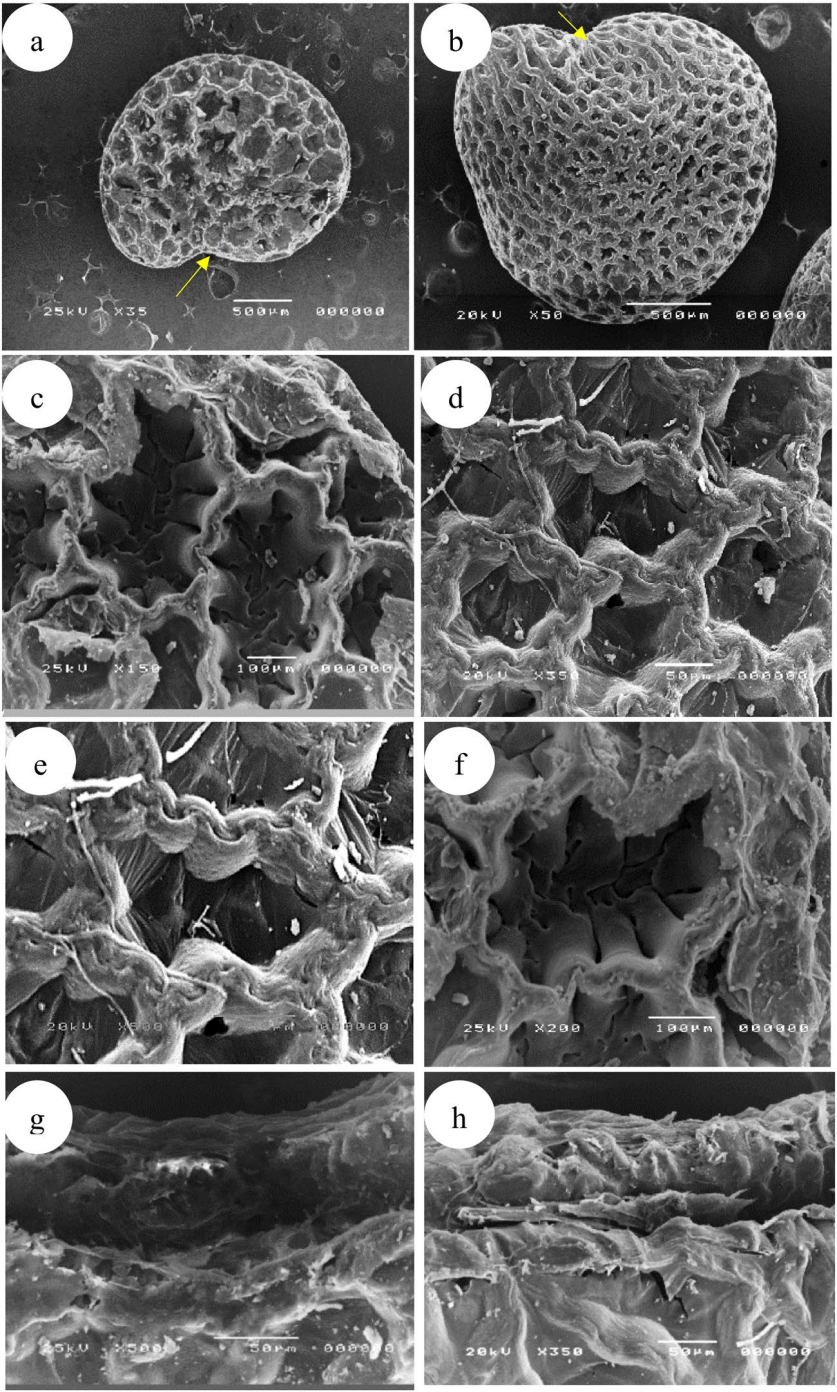
SEM photomicrographs of the seeds of *Withania*: seed shape with hilum: (**a**) *W. obtusifolia*, (**b**) *W. somnifera*; Magnified spermoderm surface: (**c**, **f**) *W. obtusifolia*; (**d**, **e**) *W. somnifera*; hilum shape: (**g**) *W. obtusifolia*, (**h**) *W. somnifera.*

*W. obusifolia* confined to Sinai, Gebel Elba characterized by rather small seeds (1.6)1.8–2.2 × 1.6–2(× 10^3^) mm drying to brown with polygonal (7–10-gonal) or oblong large cells. The basal portion of the anticlinal wall (lumen) was relatively thick with characteristic holes, smooth over the entire inner surface of the cell, and a broad ribbon-like appendage, finely papillate at the summit of the cell. While the cosmopolitan *W. somnifera* is characterized by large seeds (2.3-) 2.5–3 × 1.9–2.5 (× 10^3^) mm drying to brown with polygonal (5–6-gonal) small cells. The basal portion of the anticlinal walls is strongly thickened, the thickness covering the whole bottom, densely papillate, and fibrils present over the lateral inner surface of the cells. At the summit of the cell slender ribbon-like appendages were observed in Table 3 and Fig. 4.

The variability in seed surface patterns is seemingly very useful in the recognition of the studied species^79,80^. SEM analysis of the seeds in the present study showed that *W. somnifera* had a deeply protruding hilum, whereas *W. obtusifolia* had a shallow, slightly protruding hilum, In *W. obtusifolia*, the relief of the cell boundary is narrow and ribbon-like, with small papilla at the cell's summit; in *W. somnifera*, it is broad and densely papillate, outer periclinal wall is concave and has characteristic perforations through the thickening and lateral wall without fibrils in *W. obtusifolia* while the bottom area is covered by concave thickenings and lateral wall with fibrils in *W. somnifera*. In brief many spermodermal features are proven to be the diagnostic features that can be used to distinguish *Withania* species which agree with^80^.

#### Stem anatomy

**Fig. 5:**
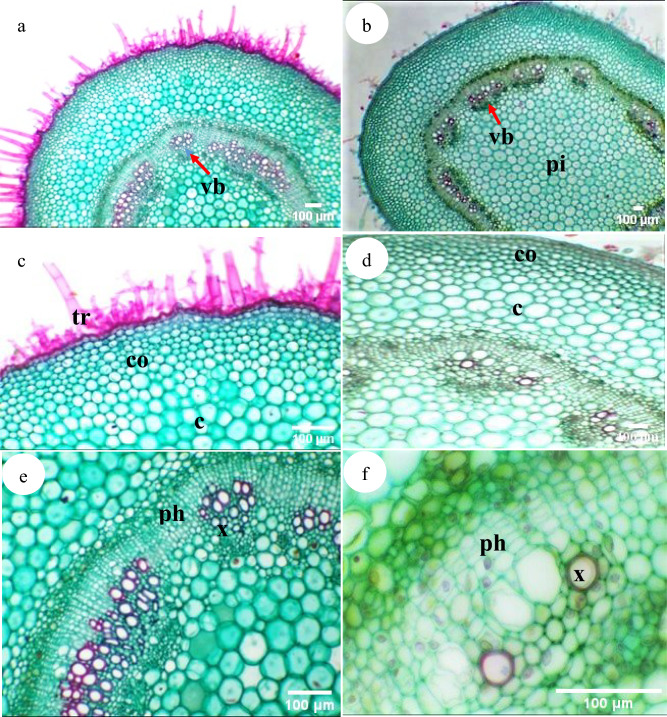
Transverse section of stem in *Withania* species. (**a, c, e**) *W. obtusifolia*, (**b, d, f**) *W. somnifera*. Abbreviations: c: cortex, co: collenchyma, p: phloem, pi: pith region, vb: vascular bundle, x: xylem.

In cross-sections, the outline of the stems is more or less circular to angular, with moderate-densely hairy. The epidermis is composed of a single layer of isodiametric-radially elongated cells covered by a very thin wavy cuticle. The cortex differentiated into 4–5 outer collenchymatous composed of isodiametric-tangentially elongated cells, followed by inner parenchymatous with isodiametric cells, filled with sand crystals. The vascular bundles are bicollateral with outer and inner phloem. Xylem with 2–5 vessels mainly in radial groups of 5–7 arches. A large central pith, composed of 16–25 layers with hexagonal and mostly isodiametric parenchymatous cells present in the center, occasionally sand crystals are present (Table 3 and Fig. 5).

#### Petiole anatomy

The outline is more or less oval with a shallow indentation in the adaxial portion. Epidermis formed of minute radially elongated cells, covered with an evident undulating cuticle and glandular, non-glandular (multicellular, and dendritic) hairs.

**Fig. 6:**
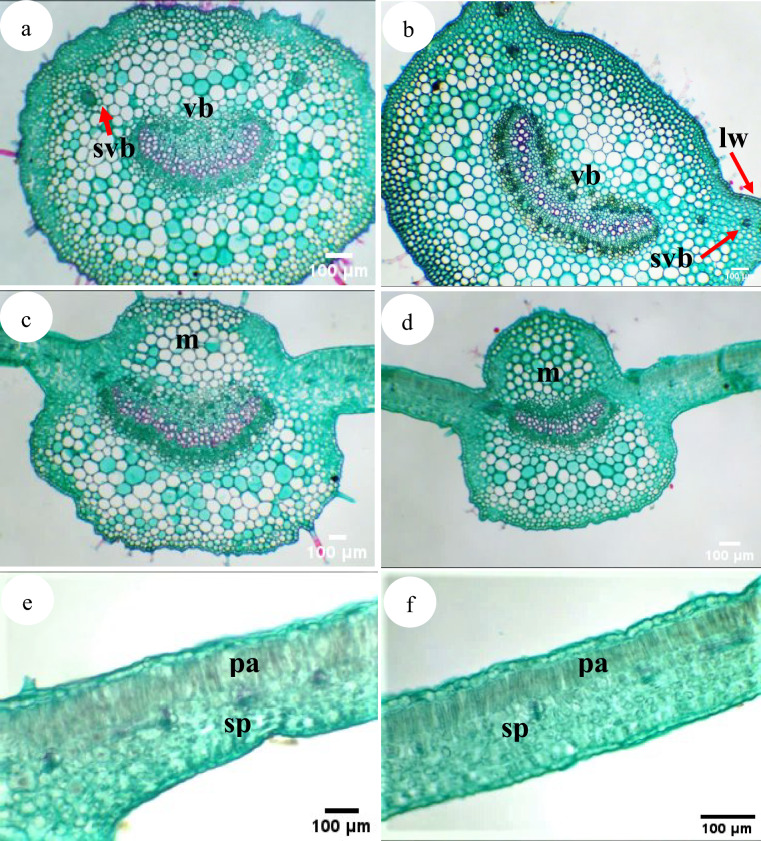
Transverse section of the petiole and the leaf in *Withania* species: (**a**, **b**) The petiole in (**a**). *W. obtusifolia*, (**b**) *W. somnifera*; (**c**, **d**) The midrib in (**c**). *W. obtusifolia*, (**d**) *W. somnifera*; (**e**, **f**) The mesophyll region in (**e**). W. *obtusifolia*, (**f**) *W. somnifera.* Abbreviations: lw: leaf wing, m: midrib region, pa: palisade tissue, sp: spongy tissue, vb: vascular bundle, svb: subsidiary vascular bundle.

Stomatal pores on both epidermal tissue facing loose parenchyma. The cortex is differentiated into outer and inner regions; the outer collenchymatous layers are formed of isodiametric-radially elongated cells and inner parenchymatous layers with radially-irregular cel. The vascular system is represented by a central elongated, arc-shaped bi-collateral vascular bundle, in which the xylem arches are ± 35, each in 2–6 series, in addition to 1–4 accessory vascular bundles at each end of the main vascular bundle (Table 4 and Fig. 6).

**Table 4:** The anatomical characteristics of the stem, leaf and petiole of the examined *Withania* species.

Characters	*W. obtusifolia*	*W. somnifera*
Stem		
outline	± circular	± circular with shallow ridges and furrows
diameter (µm)	2271–2582	2081–2804
cuticle thickness (µm)	0.51–0.64	0.5–0.55
Hairs	*Dense*	*Moderate*
Epidermal cell thickness (µm)	10.36–15.55	20–22
cortex		
Collenchyma Thickness (µm)	124.36–155.45	103.6–124.36
Parenchyma layers		
Thickness (µm)	310.89- 362.71	206–208
Number of layers	6–8	4–5
Vascular bundles		
Number of vascular bundles	14–15	17–19
External phloem		
Thickness (µm)	51.82–72.6	41.45–51.82
Number of layers	5–7	4–5
Cambium		
Thickness (µm)	20.73–41.45	31.09–41.45
Number of layers	5–6	4–5
Xylem thickness (µm)	124.36–176.17	155.45–176.17
Internal phloem		
thickness (µm)	51.82–72.6	41.45–51.82
number of layers	5–7	4–5
Pith		
diameter (µm)	880.86–932.67	880.86–1450.82
number of layers	16–17	21–25
Petiole		
Outline	*ovate*	*cat-face*
Thickness (µm)	1732.12–1856.95	1761.75–1847.847
cuticle thickness (µm)	0.5–0.6	0.5–0.6
Presence of leaf-wings	*Absent*	*Present*
Number of subsidiary vascular bundles	2–3 (1 or 2 in the each side)	3–4 (2 in each side)
Upper epidermis Thickness (µm)	25.91–31.1	20–21
Upper collenchyma		
Thickness (µm)	124.36–134.72	186.53–196.897
Number of layers	3–4(5)	6–7
Upper parenchyma		
Thickness(µm)	487.06–497.42	445.97–466.34
Number of layers	6–8	6–9
Main vascular bundle		
Thickness (µm)	487–550 × 544.06	497–550 × 1119.2
External phloem		
Thickness (µm)	72.54–82.9	93.27–105.3
Number	7–8	5–6
Xylem		
Thickness (µm)	331.62–362.71	300.53–320
Number of arches	34–35	33–34
Number of series	3–6	2–4
Cambium		
Thickness of (µm)	10.37–20.73	20.73–31.09
Number of layers	3–4	3–5
Internal phloem		
thickness (µm)	72.54–82.9	82.9–93.27
number of layers	7–8	4–6
Lower parenchyma		
Thickness (µm)	518.15–528.51	466.34–476.69
Number of layers	6–8	7–8
Cell shape	isodiametric-radially elongated	isodiametric
Lower collenchyma		
Thickness (µm)	62.18–82.9	124.36–134.72
Number of layers	4–5	4–5
Cell shape	isodiametric –tangentially elongated	isodiametric
Leaf		
Midrib region		
Diameter (µm)	1310–1855 × 1761.71	1396–1452- × 1844.61
Cuticle thickness (µm)	0.45–0.51	0.45–0.51
Upper epidermis		
Thickness (µm)	8–10	4–6
Shape	radially elongated	isodiametric-radially elongated
Upper parenchyma layers		
Thickness (µm)	495–498	435.3–445.6
Number of layers	8–9	6–8
External phloem		
Thickness (µm)	51.82–72.6	41.46–51.82
Number of layers	5–7	4–5
Xylem		
Thickness (µm)	227.99–238.35	382.5–383.42
Number of arches	32–35	35–36
Number of vessels in arches	3–6	3–5
Internal phloem		
Thickness (µm)	82.96–103.6	62.18–72.6
Number of layers	8–10	6–7
Lower parenchyma		
Thickness (µm)	362.71–383.43	341.98–362.7
Number of layers	*8–9*	*5–6*
Cell shape	radially elongated-angular	isodiametric-radially elongated
Lower collenchyma		
Thickness (µm)	73–75	112–114
Number of layers	1–2	2–3
Thickness of lower epidermis (µm)	15.55–20.73	20–21
Mesophyll region		
Thickness of lamina (µm)	337–343	218–225
Upper epidermis		
Thickness (µm)	30–32	20–21
Shape	isodiametric-tangentially elongated	tangentially elongated
Palisade tissue thickness (µm)	102–103	102–103
spongy tissue thickness (µm)	185–187	80–83
spongy cells shape	*tangentially elongated*	*radially elongated*
lower epidermis thickness (µm)	20–21	16–17

#### Leaf anatomy

Leaf blade dorsiventral. Epidermis covered with wavy cuticle, 1-layered with isodiametric-radially elongated cells. The main vascular bundle is bicollateral; Mesophyll usually differentiated into palisade and spongy parenchyma; with palisade cells usually markedly elongated, 1-layered; spongy parenchyma has 4–5 layers of tangentially- radially elongated cells.

In *W. obtusifolia*, the midrib has a broad, dome-shaped adaxial part and a tangentially extended semicircular abaxial part. The vascular system consists of a broad boat-shaped bicollateral strand of xylem band and small broken patches of phloem outside xylem arc. The size of the vascular is 372–435 μm thick. But in *W. somnifera* the midrib has a broad adaxial hump and still broader, semicircular abaxial part and continuous phloem outside the xylem. The size of the vascular is 506–538 μm thick.

Leaf anatomy is bifacial, dorsiventral with a u-shaped midrib region; upper epidermal cell isodiametric-radially elongated and covered with wavy cuticle and glandular, non-glandular (multicellular, and dendritic) trichomes. Collenchyma1- 2 layers, radially elongated cells. Parenchyma tissue 6–9 layers, iso-radially elongated cells. The main vascular bundle is bicollateral; phloem 4–7 layers upper and lower xylem region and the number of xylem arches is 32–36 each with 3–6 vessels.

Under the main vascular bundles, 5–9 layers of parenchymatous tissue with isodiametric radially elongated cells. Followed the parenchymatous tissue, 1–3 collenchyma layers with isodiametric-radially elongated cells. The parenchyma that faces the stomatal pore has wide cellular space. Lower epidermal tissue isodiametric- radially elongated cells covered with wavy cuticle. In the wings region, the mesophyll is distinguished into palisade and spongy tissues, palisade 1 layer. Spongy tissue has 4–5 layers with tangentially- radially elongated cells (Table 4 and Fig. 6).

#### Trichomes characteristics

**Fig. 7:**
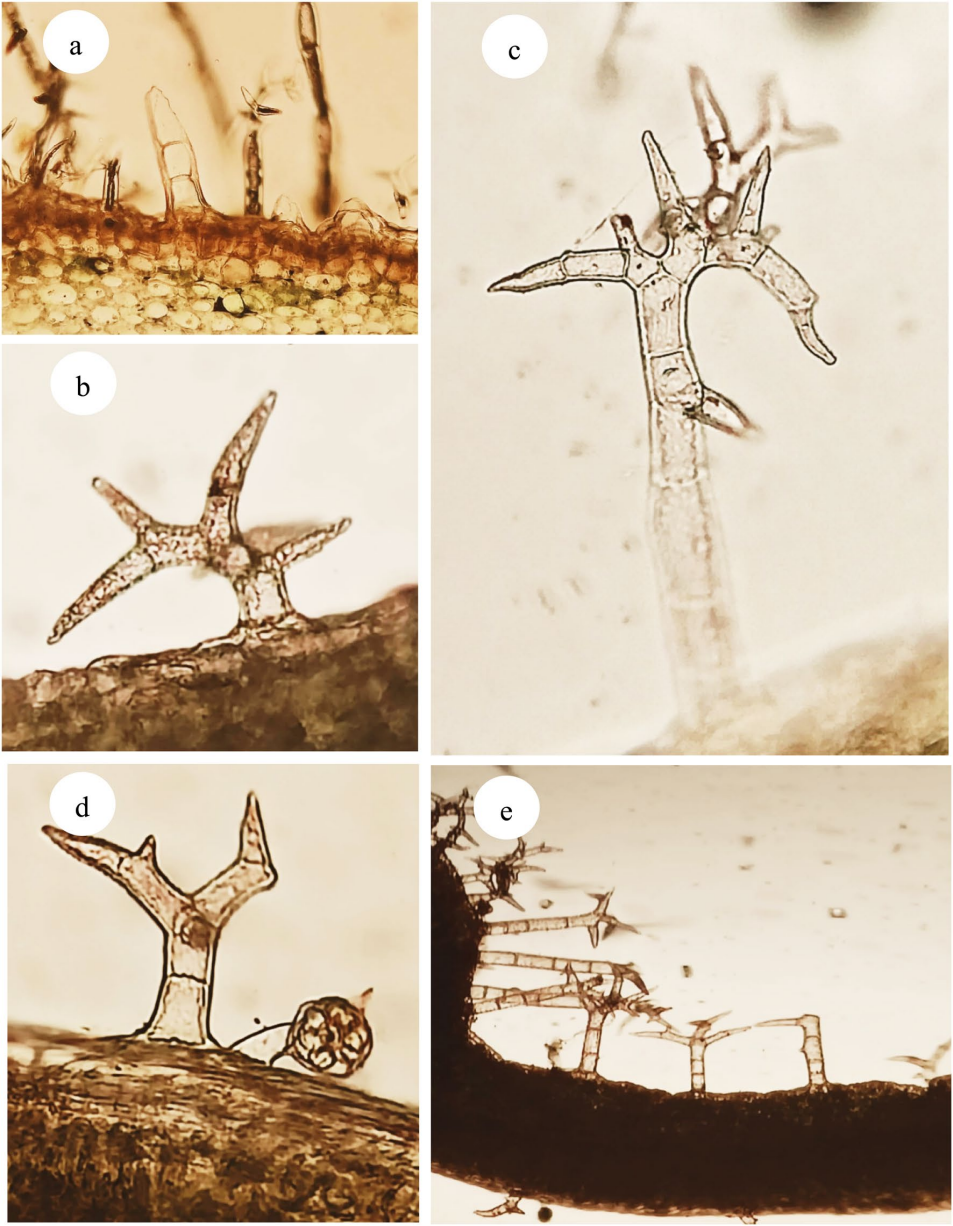
Light photograph showing morphology of individual non glandular and glandular trichomes in *Withania* species: (**a**) simple uniseriate, (**b–e**) dentritic in different forms (**e**). Glandular with multicellular head, (Magnification ×200).

Two main types of trichomes are observed on the stem, petiole, and leaf blade; non-glandular and glandular. The non-glandular trichomes can be divided into unicellular, bicellular, multicellular, and dendritic trichomes with various stalk cell numbers. The glandular trichomes with unicellular stalk and multicellular head, Fig. 7. This result agrees with^50^. The density of trichomes is more abundant in *W. obtusifolia*.

While^6^ asserts that *Withania* species differ in their morphological characteristics and^49,81^viewed the relevance of anatomical traits in distinguishing *Withania* species; stem with a circular outline in *W. obtusifolia* while circular with shallow ridges and furrows in *W. somnifera* and the number of vascular bundles; 14–15 in *W. obtusifolia* while 17–19 in *W. somnifera,* ovate petiole without ears in *W. obtusifolia* even though cat-face with ears in the other species, and leaf with tangentially elongated spongy cells in *W. obtusifolia* while radially elongated in *W. somnifera.* Other features have a limited role in characterizing the studied species which agrees with^46^ who claims that it is difficult to differentiate them by anatomical features.

#### Protein and DNA profiles

**Fig. 8:**
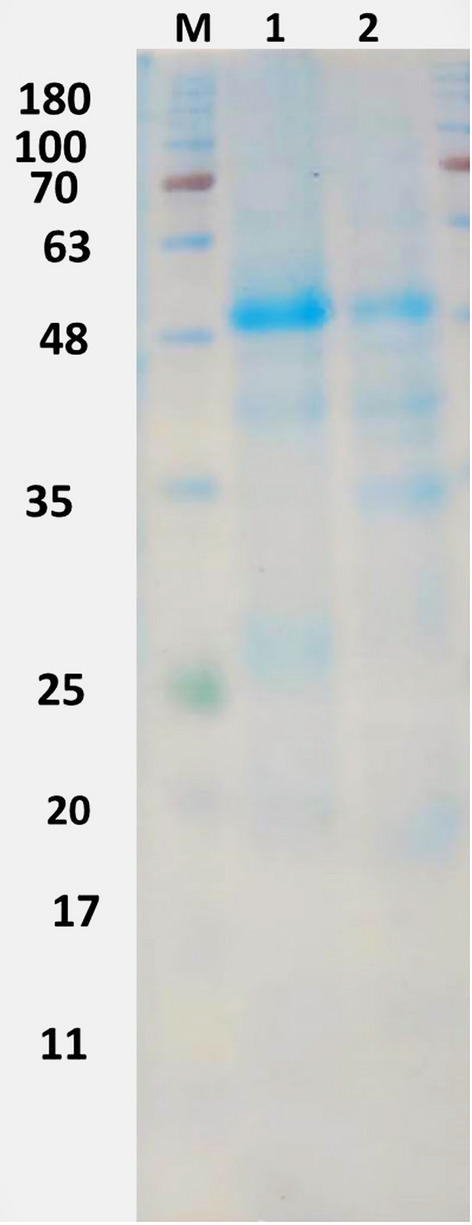
The SDS-PAGE protein profile of *W. obtusifolia* (1) and *W. somnifera* (2). Marker (M) 180 kDa.

**Fig. 9:**
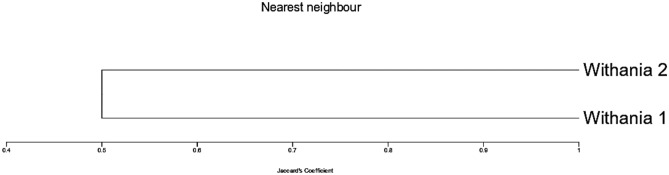
SDS-PAGE dendrogram showing the divergence between *W. obtusifolia* (1) and *W. somnifera* (2).

The SDS-protein patterns of *Withania obtusifolia* and *Withania somnifera* allowed the identification of 5–7 major bands per lane, within molecular weights (Mwt) ranging from 20 to 67 Da (Table 5 and Fig. 8). Table 5 represented the Mwt, and the rate of flow (RF) of the formed bands of the two *Withania* plants. The highest Mwt (> 60 Da) and the lowest Mwt proteins (20 Da) were recorded in both plants. Five protein bands appeared to be conserved in both plants. The highest amounts of protein bands were recorded in *W. obtusifolia* showing the appearance of two new protein bands (at about 34 Mwt and 47 Mwt). Similarities and dissimilarities of protein bands between the two *Withania* plants were inscribed in Table 6. Results revealed that *W. obtusifolia* differed from *W. somnifera* by inducing 28.57% polymorphism. The SDS-Dendrogram (Fig. 9) clarified the subordination relationship of the proteins obtained from the two *W. somnifera* plants. SDS-Dendrogram declared that the Jaccard’s similarity coefficient of the cluster analysis of the 2 plants was 0.5, which indicated that *W. obtusifolia* was moderately related to *W. somnifera*.

**Table 5:** The SDS-PAGE pattern of *W. obtusifolia* and *W. somnifera* showing molecular weight (Mwt) and the rate of flow (RF) of the formed bands.

Bands	*W. obtusifolia*		*W. somnifera*	
	Mwt	RF	Mwt	RF
1	66.54	0.17	65.80	0.18
2	53.93	0.25	51.91	0.26
3	47.14	0.29	42.57	0.33
4	42.68	0.33	26.29	0.55
5	34.47	0.42	20.00	0.69
6	26.35	0.55		
7	20.00	0.71

**Table 6:** Protein band similarities and dissimilarities prompted by *W. obtusifolia* and *W. somnifera.*

Samples	*W. obtusifolia*	*W. somnifera*
Total number of bands	7	5
Monomorphic bands	5	5
Polymorphic bands	2	–
Percentage Monomorphism	71.43	100
Percentage Polymorphism	28.57	–

**Fig. 10:**
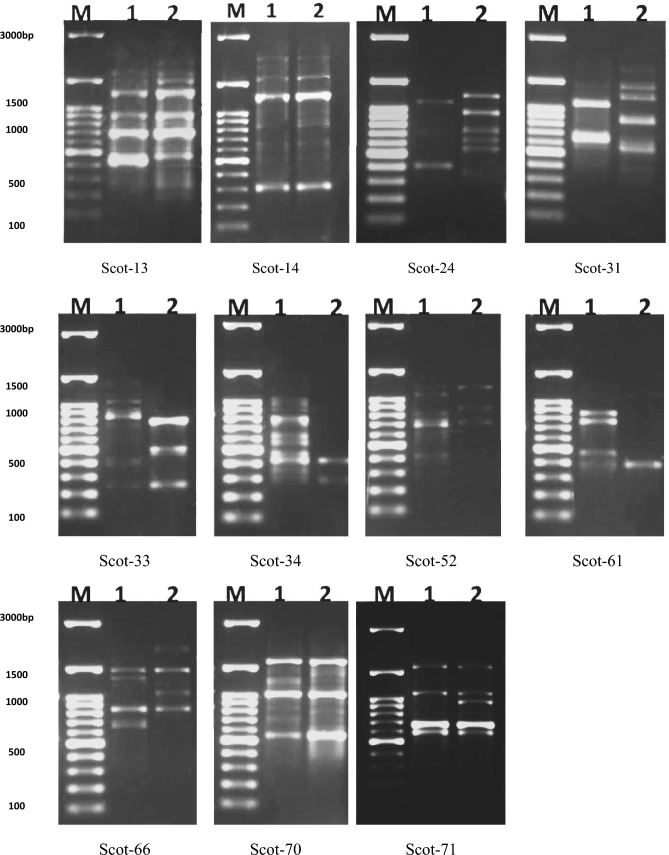
Scot-DNA banding pattern of *W. obtusifolia* (1) and *W. somnifera* (2), using eleven primers. Marker (M) 3000 bp.

**Fig. 11:**
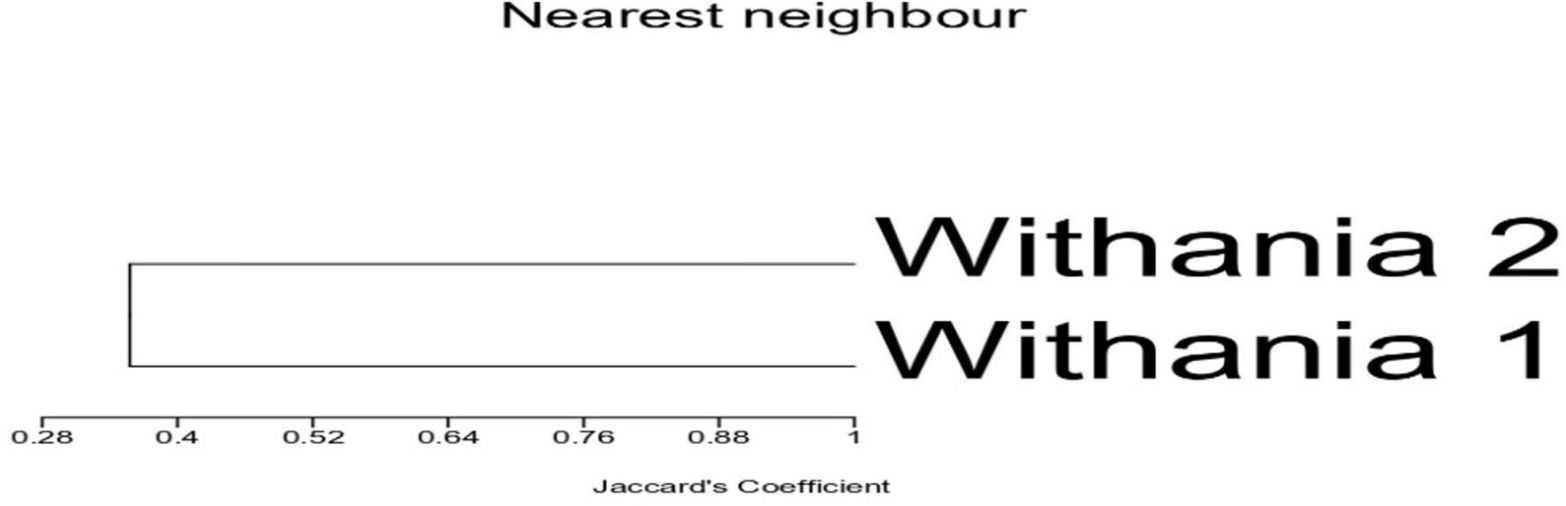
Scot-DNA-Dendrogram of *W. obtusifolia* (1) and *W. somnifera* (2) using eleven Scot-primers.

Scot-DNA analysis was used to evaluate and compare the genetic variation of the two plants and was summarized in Tables 7 and 8 and Fig. 10. Table 7 revealed the generation of 96 total bands via the usage of 11 primers with an average of 70.83% polymorphism per primer. Scot-24, Scot-31, and Scot-52 primers possessed complete discrimination ability between the two *Withania sp*. Also, Scot-70 and Scot-34 primers revealed the highest differentiation potentiality with 9 (81.82%) and 8 (80%) polymorphic bands, respectively. However, Scot-71 has the lowest discrimination ability as it exhibited 4 polymorphic bands (40%) between the plants. In addition, the other employing primers produced special banding patterns ranging from 3–7 amplicons [3 (using Scot-61), 4 (using Scot-66), 5 (using Scot-33), 6(using Scot-13), and 7 (using Scot-14)]. Similarities and dissimilarities of Scot-DNA-bands between the two plants were elucidated in Table 8 and Fig. 10. Results revealed that *W. obtusifolia* generated more polymorphic bands than those of *W. somnifera*, recording 36 (72%) and 32 (69.57) polymorphic bands, respectively. Also, both *Withania* plants owned the same conserved monomorphic bands (14). The Scot-DNA-Dendrogram illustrated the hierarchical relationship of the DNA-banding obtained from the two plants via 11 primers, Fig. 11. The Dendrogram manifested that the Jaccard’s similarity coefficient of the cluster analysis of the two plants was 0.357, which indicated that *W. obtusifolia* was poorly related to *W. somnifera*.

**Table 7:** Scot analyses of *W. obtusifolia* and *W. somnifera* using eleven primers.

Primers name	Total no. of bands	Monomorphic bands	% Monomorphism	Polymorphic bands	% Polymorphism
SCOT-13	10	4	40	6	60
SCOT-14	13	6	46.15	7	53.85
SCOT-24	8	0	0	8	100
SCOT-31	6	0	0	6	100
SCOT-33	7	2	28.57	5	71.43
SCOT-34	10	2	20	8	80
SCOT-52	8	0	0	8	100
SCOT-61	5	2	40	3	60
SCOT-66	8	4	50	4	50
SCOT-70	11	2	18.18	9	81.82
SCOT-71	10	6	60	4	40
Total	96	28	29.17	68	70.83

**Table 8:** Scot band similarities and dissimilarities prompted by *W. obtusifolia* and *W. somnifera.*

Samples	*W. obtusifolia*	*W. somnifera*
Total number of bands	50	46
Monomorphic bands	14	14
Polymorphic bands	36	32
Percentage Monomorphism	28	30.43
Percentage Polymorphism	72	69.57

In the present study, SDS-protein results revealed that *W. obtusifolia* had both conserved and additional proteins in its profile, whereas *W. somnifera* had only conserved proteins (Tables 5 and 6). Scot-DNA analyses confirm the same protein trend for DNA that *W. obtusifolia* produced more polymorphic bands while retaining monomorphic ones (Tables 7 and 8).

According to^41^, there are intraspecific variations and polymorphism phenomena in Solanaceae. Because of their coexistence in a mixed population, the two morphologically similar species *W. obtusifolia* and *W. somnifera* were frequently misinterpreted.

Genetic studies are essential for studying inter and intra-species genetic variability, in which the use of protein profiles and molecular markers are powerful and useful tools. Different studies worked on assessing the genetic variation of *W. somnifera* plants. Indian *W. somnifera* plants were genetically assessed using SDS-PAGE and RAPD markers^82^, RAPD and AFLP markers^83^, RAPD and ISSR^57,84^, RAPD^55,85,86^, ISSR^87,88^ and EST-SSR^89^, however; the Egyptian genotype was assessed with only RAPD marker by^90^. All those studies suggest the valuableness of using SDS-PAGE and molecular markers in detecting variation among *W. somnifera* plants. No studies were recorded on assessing the genetic variation of *W. obtusifolia* using protein and molecular markers analyses until^77^, who analyzed the interspecific relationship between *Withania obtusifolia* and *Withania somnifera* using morphological, anatomical, and phytochemical identification in association with SDS-PAGE and RAPD analyses and found considerable variations between both species. Finally, this investigation found that morphological (Table 3 and Figs. 1, 3, 4), and anatomical (Table 4 and Figs. 5, 6) identifications of the two species highlight slight variation between these two species, while protein (Tables 5, 6 and Fig. 8), molecular analyses (Tables 7, 8 and Fig. 10) and phylogenetic analyses (Figs. 9, 11) showed considerable genetic variations between these two species. Those findings confirmed the whole divergences found between *W. obtusifolia* and *W. somnifera*.

## Conclusion

In highlight of geographical, morphological, anatomical, pollen, and seed characteristics, the SDS-PAGE and Scot-PCR analyses of *W. obtusifolia* and *W. somnifera* confirmed the significant genetic divergence between these two species highlighted by their taxonomic analyses. Therefore, this study provided evidence that SDS-PAGE and Scot-PCR-based molecular analyses can be used as efficient tools for detecting and confirming similarity and phylogenetic relationships among the genus *Withania*.

## Data Availability

No data were taken from any database. All data generated or analyzed during this study are included in this published article. Supplementary information files during the current study available from the corresponding author on reasonable request.
